# Abnormalities of Labor

**Published:** 1881-07

**Authors:** L. B. Wells

**Affiliations:** Utica, N. Y.


### ABNORMALITIES OF LABOR.

L. B. Wells, M. D., Utica, N. Y.

Normal labor may be regarded as the accomplishment on the part of the mother of the terminal act in the process of reproduction.

When the normal period of gestation, varying from 275 to 290 days, averaging 280 days, has arrived, then the natural labor is the consummation of all that process which introduces a human being to a new existence.

The normal conditions and process of labor are so familiar to all that its discussion here may be considered superfluous.

This paper will, therefore, be limited to observations upon those abnormal conditions which are often attended with danger, and possibly the life of the mother.

Deviations from the natural presentation rendering labor more difficult, will constitute a subject for our notice.

When the head of the child presents in such a manner as to bring its anterio-posterior diameter corresponding to that of the pelvis, this may be considered normal labor.

Deviations from this in a greater or less degree may be considered as abnormal.

---

It is not necessary in this paper to enumerate these varieties, but only consider what is of more importance, whether anything can be done to avoid these sometimes difficult and perplexing conditions.

Much will depend upon the care of the patient in regard to exercise, physical exertion, etc. Bathing with tepid water in place of cold bathing, as cold bathing will be followed by a re-action tending to congestion, and mechanical support if necessary.

The question may be asked, can any remedial means be used to avert those conditions?

Some thirteen or fourteen years since I read in a homœopathic medical journal the experience of a physician, in the use of *Pulsatilla* in such cases.

From that period, in every case under my care, I have prescribed *Puls.* daily, two weeks before confinement, estimating the time from the first indications of motion of the fœtus.

In every case thus treated, the presentation has been normal, where I have been present in the early stage of labor.

I have observed one important fact, that those false manifestations do not appear, and when labor commences it goes steadily forward to its consummation.

If the physician can be thus relieved of those repeated calls, it is a very desirable relief.

It is not our province to discuss the criminal record, but only some of those accidents which are often unavoidable, and premature labor is the result.

The physician should bear in mind one fact, that every case of miscarriage predisposes to a repetition; and the more frequently it occurs, the more its probabilities are increased.

From the observation of the writer, ladies of a tall, slender form, with lax muscular fibre, are more liable to these accidents.

Can any remedial or mechanical means be available to prevent such accidents?

In answer to this, I will relate one case, as a sample of many. In 1854, I was called to attend a lady in premature labor, she being six and a half months gone. Her first child was living; she informed me that she had had five successive miscarriages, occurring from fifth to seventh month, and that her physician informed her that it would be impossible for her to have a living child.

I assured her that the doctor labored under a mistake.

Some months afterwards, I was consulted by the patient for advice, in order to prevent another mishap.

Her household duties required much constant care and motion up and down stairs. I suggested mechanical support, and gave her a vial of *Sabina*$^6$, the potency I then used; she was to take it once a day for one week, omit a week and take again the next week, in the same manner; this was to be continued to the full period of gestation. She was delivered of a fine boy, who has grown to manhood, and is now engaged in a responsible and lucrative business.

